# Characterization of the Quality and Flavor in Chinese Sausage: Comparison Between Cantonese, Five-Spice, and Mala Sausages

**DOI:** 10.3390/foods14111982

**Published:** 2025-06-04

**Authors:** Xuemei Cai, Yi Zeng, Kaixian Zhu, Yiqin Peng, Pei Xv, Ping Dong, Mingfeng Qiao, Wenjiao Fan

**Affiliations:** 1 Cuisine Science Key Laboratory of Sichuan Province, Sichuan Tourism University, Chengdu 610100, China; cxm121517@163.com (X.C.); 13378253273@126.com (K.Z.); 18780199069@163.com (Y.P.); 2 Collage of Culinary and Food Science and Engineering, Sichuan Tourism University, Chengdu 610100, China; zy13350196580@petalmail.com (Y.Z.); xupei386@gmail.com (P.X.); 0001277@sctu.edu.cn (P.D.)

**Keywords:** gas chromatography–ion mobility spectrometry (GC-IMS), free amino acids, Cantonese sausage, Five-Spice sausage, Mala sausage

## Abstract

With the increasing popularity of traditional Chinese sausages both domestically and internationally, the flavor characteristics of sausages have become an important topic in food science research. However, comparative studies on the flavor differences between different types of traditional Chinese sausages are still limited. This study aimed to systematically compare the flavor profiles of three representative types of traditional Chinese sausages (Cantonese, Five-Spice, and Mala sausages), with 20 samples randomly selected from one batch of 100 sausages per type produced in December 2024, using a combination of headspace gas chromatography–ion mobility spectrometry (HS-GC-IMS), amino acid analysis, electronic sensory analysis, and sensory evaluation techniques. Sensory evaluation revealed that Mala sausage exhibited a strong and numbing flavor. Cantonese sausage was characterized by sweet and alcoholic notes, while Five-Spice sausage displayed a more subtle and gentle flavor profile. A total of 39 volatile compounds were identified, with 2-methyl-1-butanol, 2-butanone, and butanal being the most abundant across all samples. Orthogonal partial least squares discriminant analysis (OPLS-DA) further pinpointed (+)-limonene, (Z)-ocimene, α-terpinene, β-myrcene, β-pinene, γ-terpinene, 2-pentanol, 2-octanone, and 1-hexanal as the key differential compounds responsible for the distinct flavor characteristics of each sausage type. Additionally, the free amino acid content in Mala sausage was significantly higher than that in the others, with glutamic acid and proline playing pivotal roles in shaping the taste profiles. These findings provide valuable theoretical and technical insights for the identification and control of flavor in sausage production, offering a scientific basis for guiding consumer preferences in sausages’ selection.

## 1. Introduction

Sausages, as a traditional and widely appreciated category of meat products, are renowned for their diverse flavors and extended shelf life due to low water activity and the use of flavorings [1,2]. In China, sausages have a long history and are regarded as a prized example of traditional fermented meat products. Cantonese, Five-Spice, and Mala sausages are classic types of Chinese sausages, each reflecting the distinct regional flavor profiles they represent. Cantonese sausage, celebrated for its sweetness and alcoholic fragrance derived from sugar and wine, is particularly cherished in Guangdong Province [3]. Five-Spice sausage, characterized by its signature blend of fennel, Sichuan peppercorns, star anise, cinnamon, and cloves, exhibits a mild flavor profile complemented by a mellow mouthfeel. Mala sausage, known for its pungent and numbing qualities from the addition of chili and Sichuan peppers, is primarily produced in southwestern regions of China, such as Sichuan and Chongqing [4]. As cultural exchange and technological advancements blur regional boundaries in sausage production, consumers now have access to a wide variety of flavors, yet there is still a lack of scientific understanding regarding sausage flavor profiles.

Flavor characteristics, including taste and aroma, are critical determinants of consumer preference for sausages [2,5]. Consequently, the molecular characterization of flavor compounds has become essential for developing premium-quality products. Volatile compounds (e.g., aldehydes, acids, and esters) form the aromatic foundation of fermented sausages, whereas non-volatile components such as free amino acids (FAAs), nucleotides, and organic acids primarily govern taste profiles [6]. The formation of these flavor-active compounds arises from complex metabolic processes during production, including carbohydrate fermentation, protein hydrolysis and oxidation, and lipid degradation [7]. Significant variations in flavor profiles exist among sausage types due to differences in ingredients, seasoning blends, and processing techniques. For instance, smoked sausages are generally preferred over oil-based varieties owing to their appealing color, balanced spiciness and saltiness, and reduced fat content [8]. Yin et al. [9] demonstrate that traditional Harbin red sausages contain significantly higher levels of aldehydes, ketones, phenols, and furans than their conventional counterparts, highlighting the profound impact of processing methods on the flavor of sausages. Additionally, regional sausages from different areas of Korea exhibit distinct aroma and taste profiles [10]. Similarly, Chinese dried sausages from Guangdong, Sichuan, and Harbin show notable differences in volatile compound composition, with Harbin-style sausages exhibiting the highest overall concentrations [11]. Despite growing research on sausage flavors, integrated analyses combining sensory evaluation, intelligent sensing (e.g., E-nose, E-tongue), and molecular techniques (e.g., HS-GC-IMS, amino acid analysis) remain limited for traditional Chinese sausages. This gap hinders understanding of how regional spice blends and processing methods create distinctive flavors in varieties like Cantonese, Five-Spice, and Mala sausages.

This study aimed to compare the volatile and taste profiles of Cantonese, Five-Spice, and Mala sausages by integrating sensory evaluation with E-nose, E-tongue, HS-GC-IMS, and amino acid analysis. The objectives were to identify key volatile compounds and amino acids, assess their contributions to flavor differences, and establish a scientific foundation for product development that aligns with consumer preferences.

## 2. Materials and Methods

### 2.1. Samples and Treatment

Three types of traditional Chinese sausages—Cantonese sausage (A), Five-Spice sausage (B), and Mala sausage (C)—were produced by Zhouqu Lvmai Agricultural Technology Co., Ltd. (Longnan, Gansu, China) in December 2024. Lean pork and fatty pork of the Zhouqu Black Pig (Longnan, Gansu, China) in a 7:3 ratio by weight were sliced into uniform strips (50–55 mm length × 5–7 mm width × 4–5 mm thickness) using a meat slicer (detachable grinding and cutting integrated model, CHIGO Co., Foshan, Guangdong, China), seasoned as per the formulation in Table 1 (spice provided by Lanzhou Xiaozheng Seasoning Co., Ltd., Lanzhou, Gansu, China), marinated for 12 h, and stuffed into natural pig casings (3.0 cm diameter) using a sausage stuffing machine (25 L, Jiapin Electric Appliance Co., Ltd., Shenzhen, Guangdong, China), with each type comprising 100 sausages (0.25 ± 0.02 kg per sausage). Fermentation and drying were carried out in a ventilated room (daytime temperature: ~10 °C, nighttime temperature: −2–4 °C; relative humidity: 50–70%) for 20 days until the water activity reached 0.85–0.88. Following fermentation, 20 sausages of each type were randomly selected, immediately transported to the laboratory under ice-cold conditions, and stored at −60 °C (DW-60W236, Aucma Co., Ltd., Qingdao, Shandong, China) to minimize lipid oxidation during subsequent analyses. For subsequent analysis, five sausages of each type were randomly selected and homogenized using a meat grinder (S18-LA268, Joyoung Co., Ltd., Shenzhen, Guangdong, China) to ensure sample uniformity for instrumental analysis, while the remaining fifteen sausages were retained for sensory evaluation.

### 2.2. Sensory Evaluation

The sensory evaluation was conducted in the Cuisine Science Key Laboratory in Chengdu, China. A panel of 50 trained participants, consisting of 25 female and 25 male students from the Food Science and Engineering program, was selected to evaluate the sausages. Training sessions were carried out following the guidelines established by [12]. The sausages were cleaned, steamed for 30 min, cut into approximately 0.5 cm thick pieces, and coded with three-digit random numbers before being kept warm [13]. According to the protocol described by [14], 13 sensory attributes were assessed: odor (sour, sweet, meat, spices, smoked) and taste (sour, salty, bitter, umami, meat, spices, Mala, Sichuan pepper, smoked). Sensory evaluation was performed using a ten-point category scale, where 1 indicated no intensity and 9 represented high intensity. Additionally, during the blind sensory analysis, participants were asked to identify the most distinct sausage, as the identities of the samples were concealed [15]. To minimize flavor carryover between samples, participants were provided with unsalted crackers and water to refresh their palates before each tasting.

### 2.3. Electronic Nose (E-Nose) Analysis

An odor profile analysis of the sausages was conducted using an electronic nose system (FOX 4000, Alpha M.O.S., Toulouse, France) featuring an 18-sensor metal oxide semiconductor (MOS) array. The methodology was adapted from Qiao et al. [16] with minor modifications based on preliminary experiments. For each measurement, 1.5 g of homogenized sausage was transferred to a 10 mL headspace vial and equilibrated at 50 °C for 5 min. The instrument parameters were configured as follows: 180 s purge phase, followed by a 120 s sampling and analysis cycle, with 1 mL headspace injections at 500 µL/s. Each sausage type was measured in ten replicates, from which the five most reliable datasets were selected for analysis.

### 2.4. Electronic Tongue (E-Tongue) Analysis

Taste characterization was performed using an α-ASTREE electronic tongue (Alpha M.O.S., Toulouse, France), comprising a 16-position autosampler and seven ion-selective sensors: AHS (sourness), CTS (saltiness), NMS (umami), and four cross-sensitive electrodes (PKS, ANS, SCS, CPS) [17]. Prior to analysis, 50 g of homogenized sample was blended with 200 mL of deionized water, sonicated for 40 min at 30 °C, and filtered through 0.44 μm filter papers. An 80 mL aliquot of the filtrate was analyzed under standardized conditions: 120 s measurement cycles, preceded and followed by a 180 s sensor rinsing with distilled water. Each sausage type was analyzed in ten replicates, with the five most stable datasets used for subsequent analysis.

### 2.5. Free Amino Acid (FAA) Analysis

The procedure for extracting FAAs from sausage samples was adapted from [18] with slight modifications. For each type sausage, triplicate 10 g samples were homogenized with 50 mL of 9% (W/V) sulfosalicylic acid for 3 min using a homogenizer (SCIENTZ-150A, Ningbo Scientz Biotechnology Co., Ltd., Ningbo, Zhejiang, China). The mixtures were then ultrasonically extracted at 30 °C for 40 min to ensure complete extraction. Following extraction, the pH of the mixture was adjusted to 2.20 using HCL and NaOH solutions, after which the samples were centrifuged at 10,000 rpm for 10 min. The obtained supernatant was then filtered through a 0.22 μm microfiltration membrane. Free amino acid (FAA) composition was determined via an amino acid analyzer (S433D, SYKAM, Munich, Germany) under the following conditions—column: LCA K07/Li (150 mm × 4.6 mm); detection: 570 nm and 440 nm; temperature program: reactor at 130 °C; column from 38 to 74 °C; injection: 50 μL; runtime: 108 min; ninhydrin flow rate: 0.25 mL/min. Triplicate measurements were performed for each sample.

### 2.6. HS-GC-IMS Analysis

Volatile compound profiling was conducted by HS-GC-IMS (G.A.S. Instrument, Dortmund, Germany) following reference [18]. For analysis, 3.0 g samples were sealed in headspace vials and equilibrated at 50 °C for 20 min before the automated injection of 500 μL headspace gas at 85 °C. Separation was achieved on a WAX column (30 m × 0.53 mm, 1 μm; Restek, Bellefonte, PA, USA) with IMS detection at 45 °C, using high-purity nitrogen (99.999%) as carrier gas at a flow rate of 150 mL/min. The 30 min analytical program maintained the drift tube at 45 °C with a stepped mobility gradient: an initial 2 mL/min (2 min), a ramp to 10 mL/min (8 min), then to 100 mL/min (10 min), and a final 150 mL/min (10 min). Data acquisition was carried out in positive-ion mode through VOC software version 1.0.7. Compound identification integrated four analytical approaches: NIST library (2020) matching, retention index values calibrated against C4-C9 n-alkane standards, IMS database (2024) verification, and drift time alignment. Quantitative analysis normalized the peak intensity value, with five replicate measurements per sample.

### 2.7. Statistical Analysis

Data from GC-IMS were first processed using Laboratory Analytical Viewer (LAV) software (2024) and GC-IMS library search, with visualization achieved through the Reporter and Gallery plot tools. Significant differences were assessed by one-way ANOVA, followed by Tukey’s HSD post hoc test, using SPSS 23.0 (SPSS Inc., Chicago, IL, USA; *p* < 0.05). Radar chart visualization was generated using Origin 2021 (OriginLab Co., Northampton, MA, USA). The multivariate statistical analyses included the following: principal component analysis (PCA) via GenesCloud (https://www.genescloud.cn, accessed on 10 February 2025), orthogonal partial least squares–discriminant analysis (OPLS-DA) performed in SIMCA 14.1 (Umetrics, Umea, Sweden); and variable importance in projection (VIP) scores calculated using MetaboAnalyst (https://www.metaboanalyst.ca, accessed on 19 February 2025), with VIP > 1.0 considered statistically significant. Inter-variable correlations were analyzed using the Mantel test and Pearson’s correlation, conducted with Chiplot (https://www.chiplot.online, accessed on 11 March 2025), applying a significance threshold of *p* < 0.05 (two-tailed).

## 3. Results

### 3.1. Sensory Analysis

In the blind sensory analysis, participants achieved a 96.78% accuracy in distinguishing the three sausage types, confirming significant flavor divergence among Cantonese, Five-Spice, and Mala variants.

**Figure 1:**
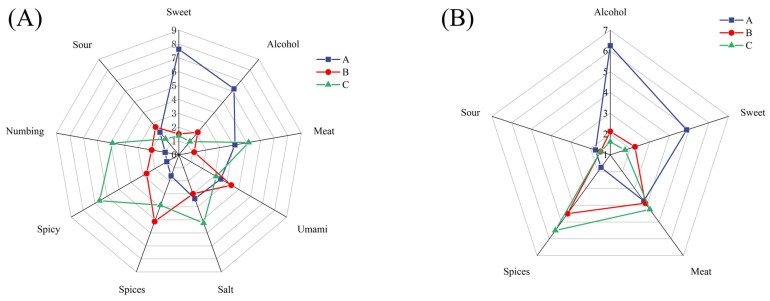
Sensory evaluation of sausages taste (**A**) and odor (**B**). A—Cantonese sausage, B—Five-Spice sausage, C—Mala sausage.

As shown in Figure 1A, the sensory scores for spicy and Sichuan pepper flavors in the Mala sausage were significantly higher than those of the other two types (*p* < 0.05). This can be attributed to the high content of capsaicinoid [19] and sanshool [20] compounds derived from chili and Sichuan pepper powders, which are known to activate the TRPV1 [21] and TRPA1 [22] receptors, respectively, inducing characteristic pungent and numbing sensations. The Cantonese sausage had significantly higher sweet and alcohol scores (*p* < 0.05), likely due to the addition of sugar and wine during processing. The Five-Spice sausage had significantly higher spice scores compared to the Cantonese sausages (*p* < 0.05). Seasoned with a blend of fennel, Sichuan pepper, star anise, cinnamon, and cloves, the Five-Spice sausage exhibited a more subtle and balanced flavor profile, characterized by mild spiciness and complex aromatic layers. There were no significant differences in umami and sour scores among the three sausages (*p* > 0.05). Notably, the meaty and salty tastes were more pronounced in the Mala sausage compared to the other two types, which is consistent with the findings of He et al. [11].

Regarding odor, the Cantonese sausage exhibited significantly stronger alcoholic and sweet odor notes compared to other variants (*p* < 0.05), which is consistent with its overall taste profile (Figure 1B). In contrast, the Mala sausage exhibited the strongest spice aroma (*p* < 0.05), primarily due to the high volatility of chili peppers, which release pungent odors rapidly, providing an immediate sensory experience [23]. Additionally, the meat aroma in the Mala sausage was relatively pronounced (*p* > 0.05). The aroma profile of the Five-Spice sausage was similar to that of the Mala sausage but with milder chili and meat notes, while its sweetness and richness were more prominent. This distinct profile can be attributed to the use of spices such as star anise and cinnamon, which have lower volatility but contribute to a more persistent aroma [24]. These findings demonstrate significant flavor variations among different sausage types, consistent with prior research documenting the substantial impact of regional ingredients and processing methods on the characteristics of Chinese sausage [25].

### 3.2. E-Nose Analysis

**Figure 2:**
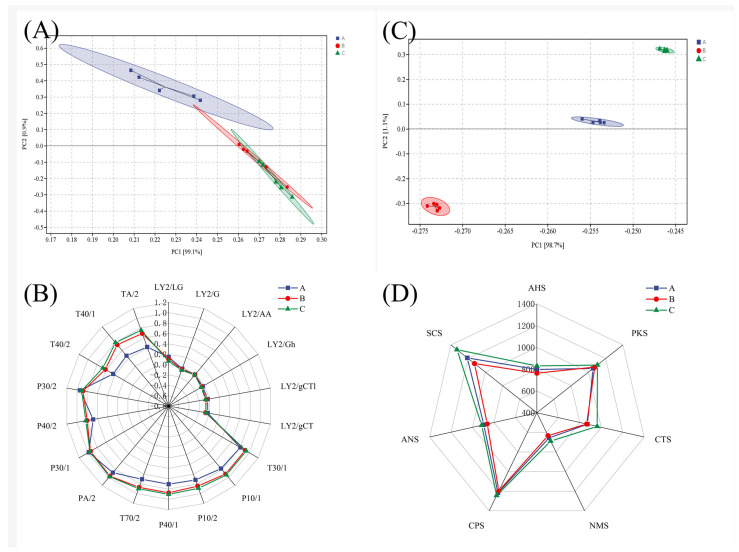
PCA (**A**) and radar chart (**B**) for E-nose data, and PCA (**C**) and radar chart (**D**) for E-tongue data of various sausage types.

E-nose analysis of the three sausage varieties revealed distinct aroma profiles. PCA demonstrated a clear separation among groups, with PC1 explaining 99.1% of the total variance (Figure 2A). Mala and Five-Spice sausages exhibited partial overlap in the PCA plot, suggesting certain shared aromatic compounds, whereas Cantonese sausage formed a completely isolated cluster, demonstrating its distinctive flavor signature.

The responses of the E-nose to different sausage types are shown in Figure 2B. Sensors sensitive to organic compounds (T30/1, P10/1, P10/2), oxidizing gases (P40/1, T70/2, PA/2), hydrocarbons (P40/2, T40/1), and aromatic compounds (T40/2, TA/2) exhibited consistent strong responses across all sausage samples [26,27]. In particular, the Mala and Five-Spice sausages showed strong and slightly different signal intensities, likely due to the presence of terpenes and aldehydes derived from chili [28] and Sichuan pepper powders [29]. This is consistent with the findings of Wu et al. [26], who reported that the addition of Sichuan pepper significantly enhanced the response of MOS sensors to these compounds in fermented chili products. The consistency between these findings suggests that the unique compounds in Sichuan pepper play a crucial role in influencing sensor responses in complex food matrices. In contrast, the Cantonese sausage exhibited lower signal intensities, consistent with its milder aroma profile, which was dominated by esters and alcohols from sugar and wine, but it showed relatively higher response values at P30/1 and P30/2, sensors that are sensitive to alcohols.

### 3.3. E-Tongue Analysis

Building on Wang’s [2] foundational work using electronic tongues for the taste profiling of dry sausages, this study extends the methodology to discriminate between three distinct sausage formulations. PCA effectively reduced the dimensionality of the electronic tongue data, achieving a clear sample classification (Figure 2C). The first two principal components (PC1: 98.7%; PC2: 1.1%) collectively explained 99.8% of the variance, demonstrating a comprehensive data representation. The distinct separation of the samples indicates notable differences in taste profiles among the sausage types.

All the sausages exhibited distinct responses across the seven electronic tongue sensors (Figure 2D). Specifically, they showed higher responses to the CPS and SCS sensors, followed by the PKS and CTS (saltiness sensor). Notably, significant differences in SCS sensor responses were observed among the sausage types. The Mala sausages showed elevated values, suggesting a richer and more pronounced flavor. This detection capability is consistent with earlier studies that validated the use of electronic sensors for resolving taste variations in dry-fermented sausages [5,30].

### 3.4. FAAs

**Figure 3:**
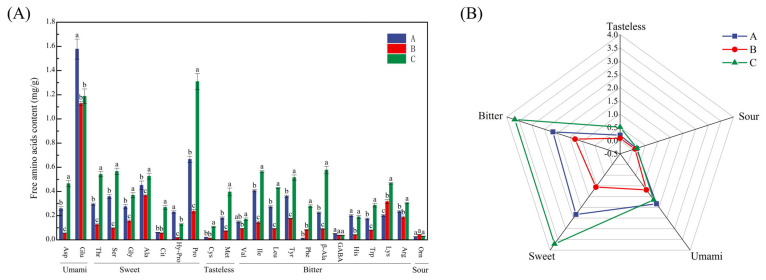
FAAs (**A**) and taste-active amino acids (**B**) in various sausage types, with lowercase letters indicating inter-sample variation, as determined by Tukey’s HSD test (*p* < 0.05).

FAAs are vital for nutrition, metabolism, and signaling, and they are also key flavor contributors in fermented sausages [31]. Branched-chain amino acids (valine, leucine, isoleucine, et al.) are specifically associated with ripened aroma formation in fermented meat products [32]. A total of 23 FAAs were identified across the three sausage types, with Mala sausage exhibiting the highest total FAA content (9.72 mg/g) (*p* < 0.05), significantly surpassing Cantonese (6.69 mg/g) and Five-Spice sausages (3.71 mg/g) (Figure 3A). This elevated FAA content in Mala sausage is likely attributed to the addition of dried chili powder, which is rich in amino acids [33].

Glutamic acid (Glu) and proline (Pro) were the predominant FAAs in all the sausages (Figure 3A, consistent with Xiao et al. [32]. Glu, a well-established umami enhancer, interacts in a positive synergistic way with other flavors, enhancing sweetness and saltiness while reducing bitterness [34]. It exhibited the highest concentration in Cantonese sausage (1.58 mg/g) (*p* < 0.05), followed by Mala (1.19 mg/g) and Five-Spice sausages (1.13 mg/g), all exceeding the umami taste threshold of 0.3 mg/g [35]. Interestingly, aspartic acid (Asp), another umami amino acid with weaker umami potential than Glu [34], was found in the highest concentration in Mala sausage (0.46 mg/g). Pro, imparting a sweet taste [36], showed the highest abundance (1.31 mg/g) among FAAs in Mala sausage, making up 13.47% of the total FAA content and surpassing Glu (12.22%). Its high concentration likely contributes to the subtle sweetness that balances the pronounced spiciness of Mala sausage.

The FAA profiles further revealed distinct taste characteristics: in Mala sausages; the levels of bitter (3.71 mg/g) and sweet (3.68 mg/g) amino acids were comparable and significantly higher than the umami amino acids (1.65 mg/g), also exceeding those found in other sausages. In contrast, Cantonese sausage showed a balanced distribution of sweet (2.33 mg/g), bitter (2.17 mg/g), and umami amino acids (1.84 mg/g), reflecting its harmonious flavor profile. On the other hand, Five-Spice sausage exhibited notably lower overall FAA concentrations (Figure 3B). These differences stem from both the composition of raw materials (e.g., chili powder, soy sauce, and spices) and the proteolytic processes during fermentation, with FAAs derived from both ingredient sources and protein hydrolysis, as noted by [37].

### 3.5. Volatile Compounds in Sausages

#### 3.5.1. GC-IMS Analysis of Volatile Compounds

**Figure 4:**
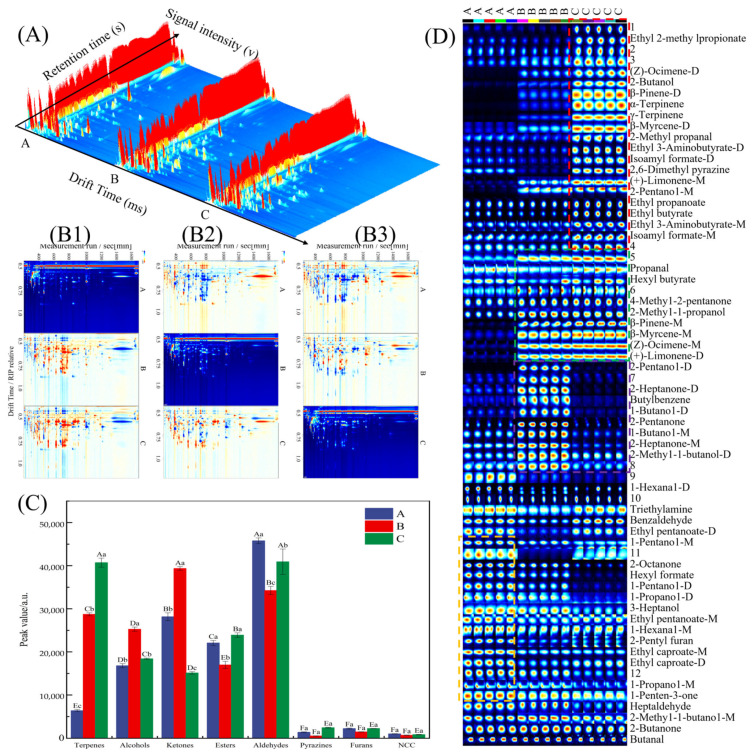
GC-IMS characterization of sausage flavor profiles: Three-dimensional map (**A**); two-dimensional maps for Cantonese (**B1**), Five-Spice (**B2**), and Mala (**B3**) as reference points, with plots of other samples compared to the reference; compound ratios (**C**), with capital letters indicating intra-sample variation and lowercase letters indicating inter-sample variation, as determined by Tukey’s HSD test (*p* < 0.05); volatile compound fingerprint (**D**), where M denotes a monomer, and D denotes a dimer.

GC-IMS was used to analyze the volatile components of different sausage types, with comparative visualization via three-dimensional and two-dimensional spectra (Figure 4A, B1, B2, B3). Figure 4A demonstrates distinct volatile profile variations among sausage types.

To better distinguish between the aromatic characteristics of the samples, the GC-IMS differential model was applied. Using Cantonese sausage as the reference (Figure 4B1), background subtraction was performed on the other samples’ topographic plots. Each point on the spectrum corresponds to a volatile compound, with color indicating signal intensity: red for high and blue for low intensity relative to the reference group. The topographic plots of Five-Spice and Mala sausages exhibited prominent red areas, indicating higher concentrations of aroma compounds compared to Cantonese sausages (Figure 4B1). When Five-Spice sausage was used as the reference, the Mala sausage showed a mixture of blue and red spots, with more red spots, indicating a richer flavor profile compared to the Five-Spice sausage (Figure 4B2).

#### 3.5.2. Identification and Comparison of Flavor Compounds

GC-IMS analysis detected 66 volatile compounds, including 54 identified through retention index (RI) and drift time (DT) matching with the library (Table 2). The total peak volume of compounds was comparable between Mala and Five-Spice sausages, but significantly lower in Cantonese sausages (*p* < 0.05), consistent with the E-nose results.

Among the 54 identified compounds, 15 were dimers; the volatile compounds included 7 terpenes, 8 alcohols, 6 ketones, 9 esters, 6 aldehydes, 1 pyrazine, 1 furan, and 1 nitrogen-containing compound (NCC). As shown in Figure 4C, aldehydes constituted the predominant volatile fraction across all sausage types, aligning with the established literature on their prevalence in fermented meat products [7]. In contrast, pyrazines, furans, and NCCs were present at lower levels. Aldehydes, formed through lipid oxidation, are known for their low flavor thresholds and contribute woody, smoky, and fatty aromas, making them essential flavor components in meat products [8]. In this study, the Cantonese and Mala sausages had significantly higher aldehyde levels than Five-Spice sausage (*p* < 0.05). Notably, Mala sausages exhibited higher terpene concentrations compared to Cantonese sausages, likely due to the use of spices such as black and white pepper, which are positively correlated with terpene levels [9]. These compounds contribute distinct herbal and citrus-like flavor profiles, as evidenced by spice sources and flavor chemistry studies [38].

Esters, alcohols, and ketones are also key flavor contributors in sausages [8]. Five-Spice sausages contained the highest ketone levels (*p* < 0.05), generated through lipid oxidation, pyruvate metabolism, and branched-chain amino acid degradation [9]. In contrast, Mala sausages exhibited lower ketone levels, a trend also seen in traditional dry sausages from Northeast China [2]. Esters levels in Mala and Cantonese sausages were much higher than in Five-Spice sausages (*p* < 0.05), whereas alcohols exhibited the opposite trend, with the highest concentrations detected in Five-Spice sausages (*p* < 0.05). This discrepancy in alcohol levels may be attributed to the inability of GC-IMS to detect ethanol, which could have influenced the observed distribution patterns.

To visualize the differences in volatile compounds, a Gallery plot was generated (Figure 4D). The columns represent volatile compounds, and the rows correspond to samples [39]. The fingerprints demonstrated the good reproducibility of the GC-IMS analysis, with compounds such as 2-methyl-1-butanol, 2-butanone, and butanal consistently present at high levels across all samples. Among these, 2-methyl-1-butanol, characterized by its sweet, fruity, and banana-like aroma, is primarily derived from wine or carbohydrate metabolism during fermentation [2]. Additionally, 2-butanone contributes sweet and fruity-like notes, while butanal imparts nutty, malty, and slightly sweet aromas, collectively enhancing the complexity of the sausage’s flavor profile.

Cantonese sausages exhibited higher concentrations of ethyl pentanoate, 2-pentyl furan, 1-hexanal, 3-heptanol, 1-pentanol, 1-propanol-D, and 1-hexanal-D (Figure 4D, yellow dotted box). These compounds contribute to the wine-like and fermented ester aromas observed in sensory evaluations [11].

In Five-Spice sausages, compounds such as 2-methyl-1-butanol-D, 2-heptanone, 1-butanol, 2-pentanone, butyl benzene, and 2-pentanol-D were found to be more abundant (Figure 4D, purple dotted box), imparting fruity and sweet aromatic notes.

Mala sausages were characterized by higher concentrations of isoamyl formate-M, butanoic acid 3-methyl ethyl ester-M, butanoic acid ethyl ester, ethyl pentanoate-D, 2-pentanol-M, (+)-limonene-M, 2,6-dimethyl pyrazine, isoamyl formate-D, butanoic acid 3-methyl ethyl ester-D, 2-methyl propanal, β-myrcene-D, γ-terpinene, α-terpinene, β-pinene-D, 2-butanol, (Z)-ocimene-D, and ethyl 2-methylpropionate-D (Figure 4D, red dotted box). Notably, γ-terpinene, (+)-limonene-M, and ethyl butyrate exhibited high signal intensities, contributing to the distinctive aroma of Mala sausages. Minor differences between Five-Spice and Mala sausages were observed for compounds such as (+)-limonene-D, (Z)-ocimene-M, β-myrcene-M, β-pinene-M, 1-propanol, 2-methyl, and 4-methyl-2-pentanone (Figure 4D, green dotted box). These compounds were present in extremely low concentrations in Cantonese sausages.

#### 3.5.3. Multivariate Statistical Analysis of Volatile Compounds in Sausages

**Figure 5:**
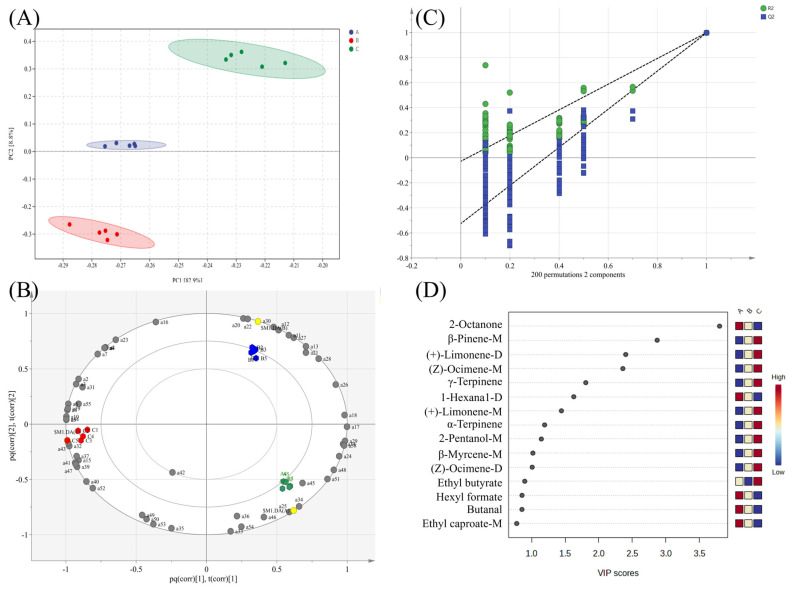
PCA score map (**A**), OPLS-DA biplot (**B**), permutation plot (**C**), and VIP diagram (**D**) of volatile flavor compounds in different sausage types.

PCA was used to examine the differences in volatile compounds among sausage types. Figure 5A shows a clear separation between groups based on peak intensity, with PC1 (82.9%) and PC2 (8.8%) collectively explaining 91.7% of the total variance. This high cumulative variance confirmed the model’s effectiveness in retaining original data characteristics [39], while the distinct grouping reflects significant differences in volatile flavor profiles among the sausage types.

To further verify these results, an OPLS-DA model was developed. The score plot (Figure 5B) shows the distinct quadrant-based clustering of the three sausage types, confirming their unique volatile profiles. The model demonstrated a strong performance, with R2X = 0.972, R2Y = 0.997, and Q2 = 0.996. To evaluate the risk of overfitting, a permutation test (*n* = 200) was performed. A 200-iteration permutation test (Figure 5C) validated the model’s robustness, with left-side Q2 values consistently below the original right-side value and the regression line intercepting the negative y-axis. A progressive Q2 decline in the stochastic models further confirmed a reliable predictive capacity for sausage differentiation [40].

#### 3.5.4. Screening and Analysis of Volatile Characteristic Markers of Various Sausage Types

VIP scores from the OPLS-DA model were used to quantify each compound’s contribution to discriminating between sausage groups. Variables with a VIP > 1 (*p* < 0.05) were deemed significant for distinguishing between samples, confirmed by permutation testing (n = 200) [18]. Based on this criterion, nine potential volatile markers were identified, including six terpenes, one alcohol, one ketone, and one aldehyde, as illustrated in Figure 5D.

The signal intensities of these markers varied significantly between the sausage types. Notably, six terpenes, (+)-limonene, (Z)-ocimene, α-terpinene, β-myrcene, β-pinene, and γ-terpinene, exhibited significantly higher levels in Mala sausages and the lowest levels in Cantonese sausages (*p* < 0.05). In contrast, 2-octanone and 1-hexanal showed significantly higher response values in Cantonese sausages (*p* < 0.05), consistent with previous studies reporting their role as key flavor compounds in traditional fermented sausages [7].

## 4. Relationship Between Sensory Evaluation, E-Nose, E-Tongue, FAAs, and VOAs

**Figure 6:**
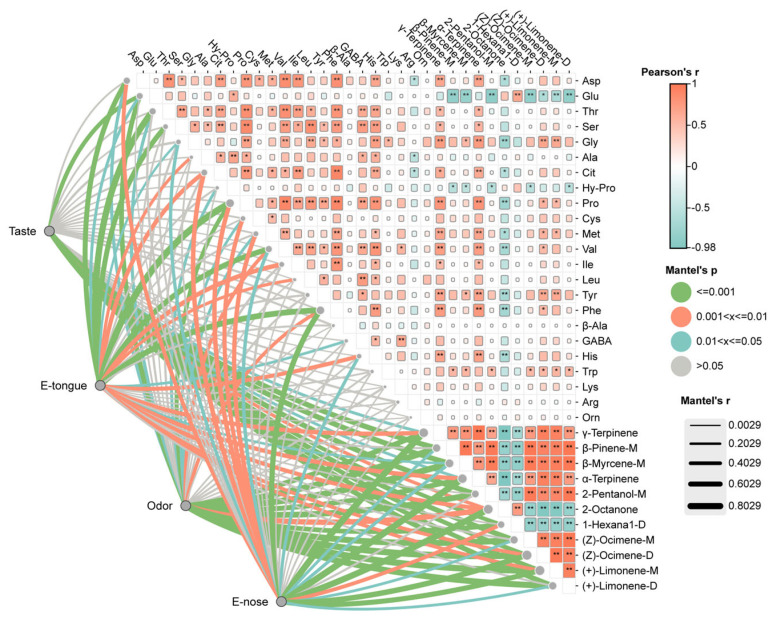
Correlation network of FAAs, volatile compounds, and sensory evaluation. * Correlation significance: * *p* < 0.05, ** *p* < 0.01.

To explore the contributions of volatile compounds and amino acids to sausage flavor and their potential interrelationships, correlation analyses were performed using Pearson and Mantel tests (Figure 6). The Pearson correlation analysis revealed strong positive correlations between γ-Terpinene, α-Terpinene, and (Z)-ocimene-D with almost all free amino acids (FAAs) (r > 0.6, *p* < 0.001). These terpenes, known for imparting fresh, herbal, and citrus notes, enhance the overall flavor profile when combined with amino acids, especially those linked to umami and savory sensations, resulting in a more balanced and fuller flavor experience [41]. In contrast, 2-octanone exhibited negative correlations with most FAAs (r < −0.5, *p* < 0.01), potentially inhibiting flavor-enhancing effects and contributing to a less desirable profile at higher concentrations.

Interestingly, Glu showed negative correlations with most volatiles except for 2-octanone and 1-hexanal-D, while Asp exhibited positive correlations with the majority of volatiles (*p* < 0.05). This divergence highlights their distinct roles, with Glu potentially stabilizing flavor and Asp enhancing aromatic complexity. The Mantel test further supported these findings, revealing significant positive correlations between sensory taste scores and Glu (Mantel’s r = 0.45, *p* < 0.001) as well as key volatiles such as γ-terpinene (Mantel’s r = 0.52, *p* < 0.001) and α-terpinene (Mantel’s r = 0.48, *p* < 0.001), indicating that Glu plays a central role in shaping the taste profile of the sausages.

E-tongue data showed significant correlations with Asp, Thr, Ser, Pro, Val, and Phe (Mantel’s r ≥ 0.6, *p* < 0.001), indicating their contributions to sweetness and bitterness [2]. Additionally, E-tongue responses correlated with γ-terpinene, α-terpinene, (Z)-ocimene-D, and (+)-Limonene-M (0.001 < *p* ≤ 0.01, Mantel’s r ≥ 0.35), suggesting their roles in modulating taste perception, likely by enhancing the complexity and balance of flavors [42]. Sensory odor scores and E-nose responses were positively correlated with all key volatiles, particularly γ-Terpinene (r = 0.68, *p* < 0.001) and α-Terpinene (r = 0.65, *p* < 0.001), underscoring their importance in defining the aromatic profile. The strong correlation between E-nose and Phe (Mantel’s r = 0.6607, *p* = 0.001) further suggests Phe’s role in enhancing aromatic intensity through Maillard reactions [43].

## 5. Conclusions

The present study provides a comprehensive understanding of the overall flavor and taste profiles of three distinct types of Chinese traditional sausages. Our findings suggest that the unique flavor characteristics of each sausage type could be leveraged in product development and regional branding. Mala sausages were characterized by a strong spicy and numbing flavor, making them suitable for consumers seeking bold, intense flavors, while Cantonese sausages emphasized sweet and alcoholic notes, appealing to those who prefer aromatic and sweet profiles. The Five-Spice sausages, with their subtle and gentle flavor, suggested a broader appeal for consumers preferring balanced flavors. The compounds 2-pentanol, 2-octanone, and 1-hexanal were identified as potential contributors to the characteristic flavor of Cantonese sausages, while (+)-limonene, (Z)-ocimene, α-terpinene, β-myrcene, β-pinene, and γ-terpinene were likely key contributors to the unique flavor of Mala sausages. Furthermore, free amino acids, particularly glutamic acid and proline, were found to significantly impact the overall taste of the sausages, with the content in Mala sausages being notably higher than in both Cantonese and Five-Spice sausages. This finding emphasizes the role of amino acids in flavor enhancement and opens avenues for optimizing taste profiles in sausage production. These results offer valuable insights for the identification and control of flavor in sausage production, providing a foundation to guide consumer preferences. However, this study was limited to analyzing three sausage types, and future research should explore other regional variations, the influence of processing methods on the development of flavor, and the interaction of key flavor compounds with other ingredients to deepen the understanding of their role in shaping the overall taste.

## Figures and Tables

**Table 1:** The ingredient lists of the sausages.

Sample	Ingredients
Mala sausage	chili powder, Sichuan pepper powder, salt, sugar, soy sauce, cooking wine, ginger, five-spice powder
Five-Spice sausage	salt, sugar, soy sauce, cooking wine, ginger, five-spice powder
Cantonese sausage	salt, sugar, soy sauce, cooking wine, ginger, white pepper powder

**Table 2:** The peak areas (mean ± sd) of molecules characterized by GC–IMS in sausages.

No.	Compounds	CAS	Formula	MW	RI	Rt [sec]	Dt [a.u.]	Peak Intensity Value/mv		
								A	B	C
a1	(+)-Limonene-D	C138863	C_10_H_16_	136.2	1210.8	744.349	1.29503	1381.73 ± 101.36 ^c^	6054.77 ± 144.94 ^b^	6821.58 ± 156.63 ^a^
a2	(+)-Limonene-M	C138863	C_10_H_16_	136.2	1210.8	744.349	1.2165	835.75 ± 27.48 ^c^	2602.98 ± 73.14 ^b^	4107.22 ± 51.94 ^a^
a3	(Z)-Ocimene-D	C470826	C_10_H_18_O	154.3	1212.3	749.263	1.72854	302.26 ± 25.22 ^c^	1444.51 ± 71.30 ^b^	2587.69 ± 115.52 ^a^
a4	(Z)-Ocimene-M	C470826	C_10_H_18_O	154.3	1211.5	746.632	1.2874	1165.85 ± 96.94 ^c^	5728.16 ± 143.35 ^b^	6508.91 ± 162.47 ^a^
a5	α-Terpinene	C99865	C_10_H_16_	136.2	1185	667.244	1.21285	131.57 ± 14.59 ^c^	662.74 ± 22.79 ^b^	2836.15 ± 140.22 ^a^
a6	β-Myrcene-D	C123353	C_10_H_16_	136.2	1168.8	630.266	1.28241	464.38 ± 16.44 ^c^	872.67 ± 5.69 ^b^	1696.69 ± 22.66 ^a^
a7	β-Myrcene-M	C123353	C_10_H_16_	136.2	1169.1	630.862	1.21729	547.26 ± 44.83 ^c^	2342.59 ± 63.78 ^b^	2856.67 ± 41.45 ^a^
a8	β-Pinene-D	C127913	C_10_H_16_	136.2	1122.8	525.358	1.64156	52.33 ± 3.49 ^c^	419.40 ± 17.42 ^b^	1347.47 ± 48.22 ^a^
a9	β-Pinene-M	C127913	C_10_H_16_	136.2	1125.9	532.452	1.22173	941.54 ± 99.78 ^c^	6463.46 ± 122.14 ^b^	7441.16 ± 157.46 ^a^
a10	γ-Terpinene	C99854	C_10_H_16_	136.2	1253.6	883.746	1.20841	197.11 ± 9.43 ^c^	1112.88 ± 16.59 ^b^	4289.87 ± 187.44 ^a^
a11	Butylbenzene	C104518	C_10_H_14_	134.2	1301.1	1036.685	1.55305	413.79 ± 16.28 ^b^	1042.79 ± 47.63 ^a^	194.57 ± 7.08 ^c^
a12	1-Butano1-D	C71363	C_4_H_10_O	74.1	1152.1	592.094	1.38008	351.63 ± 16.77 ^b^	907.42 ± 41.08 ^a^	234.38 ± 18.40 ^c^
a13	1-Butano1-M	C71363	C_4_H_10_O	74.1	1151.6	591.003	1.18211	845.36 ± 43.08 ^b^	1437.00 ± 27.07 ^a^	449.59 ± 26.02 ^c^
a14	1-Pentanol-D	C71410	C_5_H_12_O	88.1	1270.1	937.241	1.50791	602.58 ± 25.21 ^a^	461.03 ± 57.09 ^b^	163.60 ± 9.41 ^c^
a15	1-Pentanol-M	C71410	C_5_H_12_O	88.1	1269.8	936.371	1.24896	2560.00 ± 68.33 ^b^	2433.33 ± 90.68 ^c^	3078.24 ± 93.57 ^a^
a16	2-Methyl-1-propanol	C78831	C_4_H_10_O	74.1	1103.3	480.693	1.17181	1055.47 ± 32.06 ^c^	2060.64 ± 91.48 ^a^	1758.26 ± 88.98 ^b^
a17	1-Propanol-D	C71238	C_3_H_8_O	60.1	1048.1	406.778	1.25236	2631.11 ± 116.51 ^a^	2345.42 ± 43.46 ^b^	1058.19 ± 44.46 ^c^
a18	1-Propanol-M	C71238	C_3_H_8_O	60.1	1049.1	407.995	1.11553	2337.42 ± 92.51 ^a^	2281.30 ± 101.36 ^a^	1458.09 ± 19.21 ^b^
a19	2-Butanol	C586629	C_10_H_16_	136.2	1292.2	1009.333	1.21881	209.95 ± 20.42 ^c^	625.60 ± 8.25 ^b^	1609.68 ± 118.15 ^a^
a20	2-Methy1-1-butanol-D	C137326	C_5_H_12_O	88.1	1219	771.205	1.47937	1604.34 ± 81.59 ^c^	3155.10 ± 77.85 ^a^	1789.69 ± 43.06 ^b^
a21	2-Methy1-1-butanol-M	C137326	C_5_H_12_O	88.1	1218.4	769.028	1.23527	3379.02 ± 92.35 ^b^	3695.32 ± 73.21 ^a^	3132.56 ± 88.50 ^c^
a22	2-Pentanol-D	C6032297	C_5_H_12_O	88.1	1128.3	537.853	1.44523	226.92 ± 29.53 ^c^	2432.11 ± 134.72 ^a^	415.10 ± 19.74 ^b^
a23	2-Pentanol-M	C6032297	C_5_H_12_O	88.1	1127.8	536.743	1.2028	286.17 ± 28.75 ^b^	2791.81 ± 42.58 ^a^	2879.83 ± 48.30 ^a^
a24	3-Heptanol	C589822	C_7_H_16_O	116.2	1270.4	938.417	1.32364	853.40 ± 27.72 ^a^	671.10 ± 41.74 ^b^	426.99 ± 40.25 ^c^
a25	1-Penten-3-one	C1629589	C_5_H_8_O	84.1	1022	374.919	1.0843	861.75 ± 22.21 ^a^	658.41 ± 7.14 ^b^	666.85 ± 13.69 ^b^
a26	2-Butanone	C78933	C_4_H_8_O	72.1	919.8	299.424	1.24261	5066.95 ± 80.04 ^a^	5394.54 ± 104.05 ^a^	3974.76 ± 164.59 ^b^
a27	2-Heptanone-D	C110430	C_7_H_14_O	114.2	1193.2	687.111	1.63358	1325.02 ± 113.11 ^b^	3516.69 ± 105.37 ^a^	405.89 ± 51.08 ^c^
a28	2-Heptanone-M	C110430	C_7_H_14_O	114.2	1193.4	687.778	1.2581	1611.74 ± 120.39 ^b^	2353.83 ± 19.21 ^a^	798.64 ± 42.34 ^c^
a29	2-Octanone	C111137	C_8_H_16_O	128.2	1301.6	1038.18	1.33107	11,548.01 ± 683.56 ^a^	8926.33 ± 173.69 ^b^	2943.59 ± 326.65 ^c^
a30	2-Pentanone	C107879	C_5_H_10_O	86.1	998.5	346.255	1.37465	6582.67 ± 125.44 ^b^	17,218.75 ± 106.71 ^a^	4952.58 ± 206.68 ^c^
a31	4-Methyl-2-pentanone	C108101	C_6_H_12_O	100.2	1023.4	376.556	1.17907	1179.89 ± 76.30 ^c^	1368.02 ± 54.63 ^b^	1560.64 ± 37.58 ^a^
a32	Ethyl 2-methy lpropionate	C97621	C_6_H_12_O_2_	116.2	982.3	335.319	1.56858	457.42 ± 20.30 ^b^	410.31 ± 16.78 ^c^	1202.13 ± 87.16 ^a^
a33	Ethyl caproate-D	C123660	C_8_H_16_O_2_	144.2	1246	858.818	1.79845	2840.88 ± 155.85 ^a^	1635.47 ± 67.24 ^c^	2205.41 ± 113.37 ^b^
a34	Ethyl caproate-M	C123660	C_8_H_16_O_2_	144.2	1246	858.818	1.33659	5389.05 ± 207.21 ^a^	3737.74 ± 81.61 ^b^	3624.88 ± 50.89 ^b^
a35	Ethyl-pentanoate-D	C539822	C_7_H_14_O_2_	130.2	1140.2	564.987	1.67641	319.18 ± 18.13 ^a^	170.08 ± 8.96 ^b^	299.52 ± 21.97 ^a^
a36	Ethyl pentanoate-M	C539822	C_7_H_14_O_2_	130.2	1141.9	568.842	1.26702	1089.80 ± 48.93 ^a^	946.63 ± 38.65 ^b^	1004.78 ± 34.03 ^a^
a37	Ethyl propanoate	C105373	C_5_H_10_O_2_	102.1	969.9	328.166	1.44773	2058.83 ± 77.05 ^b^	1942.57 ± 61.99 ^b^	2646.72 ± 144.53 ^a^
a38	Hexyl formate	C629334	C_7_H_14_O_2_	130.2	1371.3	1247.021	1.32883	2503.58 ± 125.40 ^a^	1843.61 ± 61.36 ^b^	561.38 ± 29.67 ^c^
a39	Isoamyl formate-D	C110452	C_6_H_12_O_2_	116.2	1076.2	441.009	1.65656	417.59 ± 16.06 ^b^	259.12 ± 20.96 ^c^	886.02 ± 42.70 ^a^
a40	Isoamyl formate-M	C110452	C_6_H_12_O_2_	116.2	1079.3	444.825	1.26587	1755.11 ± 35.54 ^b^	1313.40 ± 43.31 ^c^	2448.57 ± 37.91 ^a^
a41	Ethyl butyrate	C105544	C_6_H_12_O_2_	116.2	1045.1	403.106	1.56142	2151.89 ± 65.15 ^b^	1578.53 ± 64.89 ^c^	4186.98 ± 180.72 ^a^
a42	Hexyl butyrate	C2639636	C_10_H_20_O_2_	172.3	1408.5	1358.583	1.47487	1395.45 ± 44.60 ^a^	1165.37 ± 358.99 ^a^	1451.94 ± 255.91 ^a^
a43	Ethyl 3-Aminobutyrate-D	C108645	C_7_H_14_O_2_	130.2	1059.1	420.171	1.65735	315.60 ± 13.29 ^b^	303.94 ± 22.09 ^b^	758.60 ± 42.16 ^a^
a44	Ethyl 3-Aminobutyrate-M	C108645	C_7_H_14_O_2_	130.2	1057.8	418.611	1.24472	1528.04 ± 22.94 ^c^	1724.34 ± 49.32 ^b^	2482.35 ± 47.11 ^a^
a45	1-Hexana1-D	C66251	C_6_H_12_O	100.2	1098.9	470.772	1.56793	7863.17 ± 193.38 ^a^	5146.35 ± 1059.19 ^ab^	4176.00 ± 1573.27 ^b^
a46	1-Hexana1-M	C66251	C_6_H_12_O	100.2	1099.9	473.061	1.26225	2972.14 ± 66.10 ^a^	2268.20 ± 51.31 ^b^	2427.26 ± 226.44 ^b^
a47	2-Methyl propanal	C78842	C_4_H_8_O	72.1	822.2	243.266	1.09122	1575.01 ± 64.09 ^b^	1211.87 ± 32.74 ^c^	2767.80 ± 107.16 ^a^
a48	Butanal	C123728	C_4_H_8_O	72.1	861.8	266.053	1.1196	15,649.29 ± 108.50 ^a^	14,586.89 ± 135.27 ^b^	13,708.93 ± 87.22 ^c^
a49	Propanal	C123386	C_3_H_6_O	58.1	767.7	211.985	1.04748	1967.36 ± 70.15 ^a^	1695.08 ± 34.16 ^b^	2012.86 ± 81.27 ^a^
a50	Benzaldehyde	C100527	C_7_H_6_O	106.1	1520.8	1695.416	1.14217	13,676.70 ± 366.67 ^a^	8215.54 ± 1233.37 ^b^	14,210.80 ± 1007.29 ^a^
a51	Heptaldehyde	C111717	C_7_H_14_O	114.2	1197.3	700.409	1.32526	1989.19 ± 29.67 ^a^	1510.19 ± 58.38 ^b^	1226.10 ± 66.75 ^c^
a52	2,6-Dimethyl pyrazine	C108509	C_6_H_8_N_2_	108.1	1360.8	1215.695	1.14064	1375.41 ± 109.97 ^b^	554.80 ± 42.02 ^c^	2367.37 ± 142.44 ^a^
a53	2-Pentyl furan	C3777693	C_9_H_14_O	138.2	1241.6	844.547	1.25208	2222.82 ± 106.41 ^a^	1455.03 ± 62.57 ^b^	2244.39 ± 41.95 ^a^
a54	Triethylamine	C121448	C_6_H_15_N	101.2	772.3	214.611	1.09247	979.49 ± 36.46 ^a^	689.55 ± 28.97 ^c^	804.28 ± 45.08 ^b^

Note: Different lower-case letters represent significant differences among samples as tested by Tukey’s HSD test (*p* < 0.05); CAS—Chemical Abstracts Service registry number; MW—molecular weight.; RI—retention index, calculated using n-ketones C4-C9 as external standard; RT—retention time; M—the monomer, D—the dimer; A—Cantonese sausage, B—Five-Spice sausage, C—Mala sausage.

## Data Availability

The original contributions presented in this study are included in the article. Further inquiries can be directed to the corresponding authors.
